# The shape of the healthy heart is optimized for vortex ring formation

**DOI:** 10.1186/1532-429X-18-S1-O23

**Published:** 2016-01-27

**Authors:** Per M Arvidsson, Sándor J Kovács, Johannes Töger, Rasmus Borgquist, Einar Heiberg, Marcus Carlsson, Håkan Arheden

**Affiliations:** 1 grid.4514.40000000109302361Department of Clinical Physiology, Lund University, Lund, Sweden; 2 grid.4367.60000000123557002Cardiovascular Biophysics Laboratory, Cardiovascular Division, Washington University, St. Louis, MO USA; 3 grid.4514.40000000109302361Department of Cardiology, Arrhythmia Clinic, Lund University, Lund, Sweden

## Background

**Figure 1:**
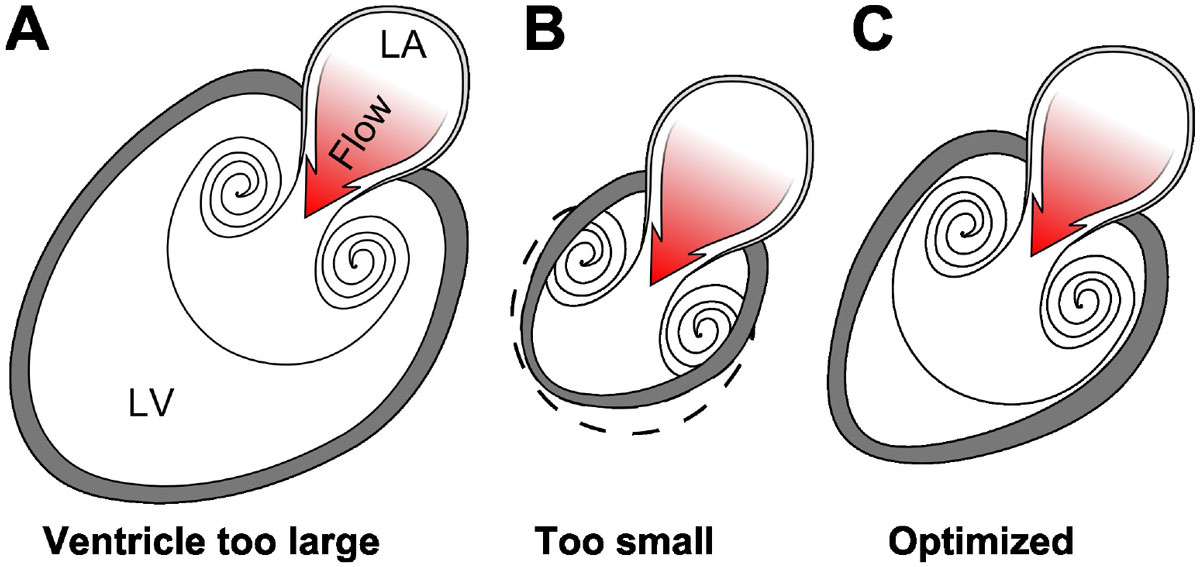
**Possible anatomical arrangements for intracardiac vortex ring generation**. **A,** Vortex rings are formed by the mitral orifice. A large left ventricle can accommodate the full size of the vortex ring it generates, but large ventricles have high wall tension which causes energy waste. **B**, If the chamber is too small it cannot accommodate full vortex ring formation, and is therefore incompatible with efficient filling. **C**, An optimized left ventricle enables energy-efficient filling by matching endocardial expansion to vortex ring formation and expansion, and maintains low wall tension.

Intracardiac blood flow is known to influence cardiac development through transduction of endothelial shear forces. Vortex rings inside the left ventricle constitute a possible "blueprint" for cardiogenesis, the hemodynamic determinant of final cardiac shape. However, the relationship between the vortex ring and endocardium has previously not been quantified, and the influence of the vortex ring dimensions on the shape of the heart has therefore not been considered. We hypothesized a dynamic coupling between the vortex ring and the healthy left ventricle throughout diastole, and uncoupling in the diseased heart (Fig. 1).

## Methods

16 healthy volunteers and 23 patients with heart failure (n = 12 ischemic, n = 11 non-ischemic dilated cardiomyopathy), underwent CMR examination at 1.5T (n = 27) or 3T (n = 8), including 4D flow. Vortex ring boundary was calculated using Lagrangian Coherent Structures (LCS) and CUDA parallel computing. Vortex ring and LV endocardium were manually delineated, and the average distance between the delineations was measured for all time points in diastole.

## Results

**Figure 2:**
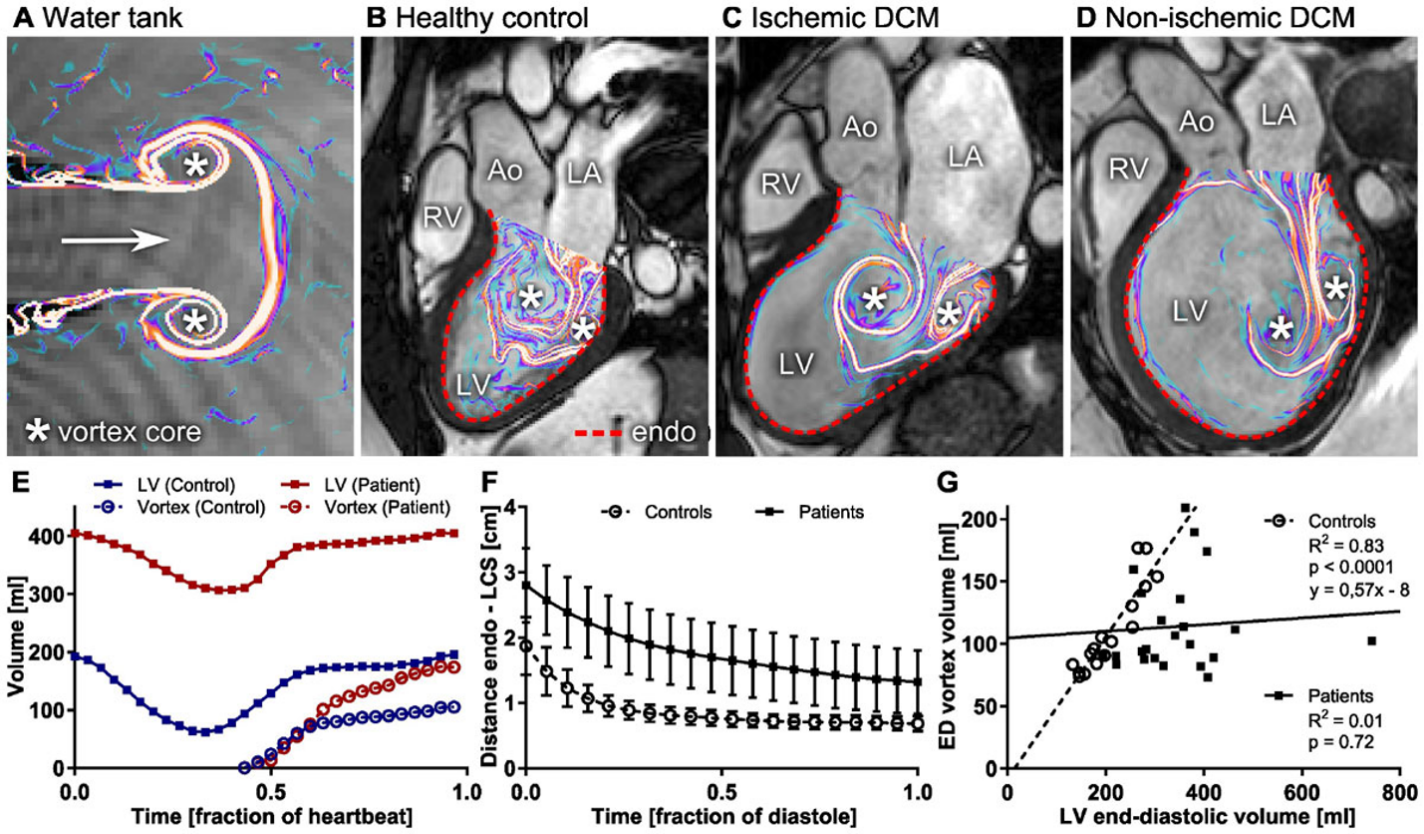
**A, Symmetrical vortex ring generated in water tank, visualized using Lagrangian Coherent Structures (LCS)**. **B**, Vortex ring formation during filling of the LV in a healthy control displays additional complexity compared to the water tank model. Note the close proximity between the vortex ring boundary and the LV endocardium. Larger distance was seen in patients (**C, D**). **E**, Volume-time curves of one control and one patient. **F,** Distance between vortex ring boundary (LCS) and endocardium of the left ventricle (mean ± SD). **G**, Vortex ring volume at end-diastole was closely correlated to LV end-diastolic volume in controls. LV chamber size is tuned to accommodate the vortex ring in healthy hearts regardless of total heart size. In contrast, patients demonstrate a loss of optimized vortex-wall coupling.

Vortex formation is shown in a control (Fig. 2B) and in two patients (Fig. 2C & 2D).

In controls, regardless of LV size, the vortex ring boundary evolved close to the endocardial border (Fig 2E & 2F). By end-diastole, the vortex ring occupied on average 53% (95% CI: 49-58%) of the total LV volume. Vortex ring volume at end-diastole was strongly correlated to LV end-diastolic volume (R^2^ = 0.83, Fig. 2G).

In the enlarged, failing heart, the vortex boundary formed at a greater distance from the endocardium. By end diastole, the vortex ring occupied on average 35% of the ventricle (95% CI: 30-41%). Vortex ring growth was not coupled to the total LV volume (Fig. 2G).

## Conclusions

The spatiotemporal dynamics of the healthy left ventricle is optimized to accommodate diastolic vortex ring formation and its evolution. Vortex rings are consistent across a wide size range of healthy hearts but significantly disturbed in heart failure, which increases our understanding of how fluid dynamics is coupled to, and governs cardiac shape and function. Vortex ring parameters carry implications for exercise physiology, cardiac surgery and design and implantation of prosthetic valves.

